# Erratum for “Isolation and Characterization of a Novel Facultative Anaerobic Filamentous Fungus from Japanese Rice Field Soil”

**DOI:** 10.1155/2010/684205

**Published:** 2010-04-14

**Authors:** Akio Tonouchi

**Affiliations:** Faculty of Agriculture and Life Science, Hirosaki University, 3 Bunkyo-cho, Hirosaki 036-8561, Japan

The incorrect deposition number of RB-1 was showed in the section Materials and Methods. The correct deposition number is JCM13870.

